# The Role of Dynamic Contrast-Enhanced Magnetic Resonance Imaging (DCE-MRI) in Differentiating Salivary Gland Neoplasms Compared with Fine-Needle Aspiration (FNA)

**DOI:** 10.3390/diagnostics16142238

**Published:** 2026-07-17

**Authors:** Weronika Oleksiuk, Wiktoria Dembowska, Mateusz Owsiak, Łukasz Zwarzany, Wojciech Poncyliusz, Kamal Morshed, Katarzyna Radomska

**Affiliations:** 1 Department of Adult and Children Otolaryngology and Otolaryngological Oncology, Pomeranian Medical University, 70-204 Szczecin, Polandkatarzyna.radomska@pum.edu.pl (K.R.); 2 Department of Otolaryngology and Laryngological Oncology, University of Radom, 26-600 Radom, Poland; 3 Department of Diagnostic Imaging and Interventional Radiology, Pomeranian Medical University, 70-204 Szczecin, Poland; mateusz.owsiak@pum.edu.pl (M.O.); lukasz.zawrzany@pum.edu.pl (Ł.Z.); wojciech.poncyliusz@pum.edu.pl (W.P.)

**Keywords:** parotid gland, dynamic contrast-enhanced magnetic resonance imaging, fine-needle aspiration

## Abstract

**Background/Objectives:** Salivary gland tumors account for 2.0–6.5% of all head and neck neoplasms, approximately 70% of which are located in the parotid gland. Preoperative determination of tumor histology is a crucial step in the diagnostic and therapeutic pathway because it guides the choice of surgical technique, affecting the achievement of adequate resection margins and preservation of the facial nerve. Fine-needle aspiration (FNA) cytology is the most commonly used cytologic method. However the complex and heterogenous histologic architecture of salivary gland tumors is associated with a significant proportion of false results. Dynamic contrast-enhanced magnetic resonance imaging (DCE–MRI) is a valuable complementary diagnostic tool that enables noninvasive analysis based on perfusion assessment and tissue characterization. The aim of this study is to evaluate the utility of dynamic contrast-enhanced magnetic resonance imaging (DCE-MRI) in the diagnosis of salivary gland tumors and to compare its diagnostic performance with fine-needle aspiration (FNA). **Methods:** We present a study of 25 patients who underwent DCE-MRI, FNA and postoperative histopathologic examination. **Results:** Among the 25 patients included, DCE-MRI results were concordant with the postoperative histopathologic diagnosis in 76% of cases, whereas only 36% of positive FNA results correlated with the histopathologic diagnosis. **Conclusions:** DCE-MRI demonstrates greater correlation with postoperative histopathologic diagnosis compared with FNA. Advantages of DCE-MRI include its noninvasive nature and the ability to image the entire region of interest. Key benefits of FNA are its wide availability and low cost. Selecting the most appropriate preoperative diagnostic modality can positively influence subsequent diagnostic and therapeutic management.

## 1. Introduction

Salivary gland tumors represent a relatively rare but clinically significant group of head and neck neoplasms, accounting for approximately 2–6% of all cases in this region [1,2,3,4,5]. The vast majority (70–80%) arise in the parotid gland, with far fewer occurring in the submandibular gland or minor salivary glands [2,6,7]. The incidence of these tumors has shown a gradual increase, particularly in aging populations, with peak occurrence in the sixth and seventh decades of life, with a slight female predominance [4,5]. Commonly reported risk factors include tobacco smoking, chronic exposure to ionizing radiation, and environmental or occupational exposures [4,8]. Incidence has increased in recent years, likely reflecting both improved diagnostic detection and a genuine rise in case numbers [5,7]. Most salivary gland tumors are benign, predominantly pleomorphic adenomas and Warthin tumors [6,9,10], which differ in histologic architecture, growth dynamics, and malignant potential [11]. Malignant lesions are much less common but display considerable morphological heterogeneity, variable clinical courses, and sometimes high aggressiveness. This heterogeneity makes accurate differential diagnosis of salivary gland tumors a significant clinical challenge that directly influences treatment strategy and prognosis [4,7]. Contemporary diagnostic workup combines imaging modalities and cytologic assessment. Fine-needle aspiration (FNA) remains the gold standard for initial cytologic assessment due to its cost-effectiveness and minimal invasiveness [12]. To standardize reporting and stratify malignancy risk, FNA results are classified according to the Milan System for Reporting Salivary Gland Cytopathology (MSRSGC). The Milan System divides cytologic diagnoses into six diagnostic categories, each associated with a risk of malignancy (ROM) and recommended clinical management. However, its diagnostic value can be limited, particularly for tumors with complex histology. Lesions classified as Category III (Atypia of Undetermined Significance) or Category IVb (Salivary Gland Neoplasm of Uncertain Malignant Potential) present a significant clinical dilemma, as they often result in indeterminate findings that may delay definitive treatment or lead to suboptimal surgical planning. In response to these cytologic limitations, advanced magnetic resonance imaging techniques, such as dynamic contrast-enhanced MRI (DCE-MRI) and diffusion-weighted imaging (DWI), have emerged as critical adjuncts [13,14]. Unlike conventional MRI, which focuses on anatomical morphology, DCE-MRI allows for quantitative and qualitative analysis of tissue microvasculature, perfusion, and capillary permeability. By evaluating the time–intensity curve (TIC) types, clinicians can differentiate between rapid washout characteristic of Warthin tumors and the progressive enhancement typical of pleomorphic adenomas. Furthermore, the integration of the apparent diffusion coefficient (ADC) from DWI sequences provides insights into cellular density, further refining the distinction between benign and malignant processes [15,16,17,18].

## 2. Materials and Methods

The retrospective analysis included 25 patients treated at the Department of Adult and Children Otolaryngology and Otolaryngological Oncology, Pomeranian Medical University, USK1 Szczecin, between 2023 and 2024 for focal salivary gland lesions. During the study period, a total of 149 salivary gland tumor surgeries were performed—81 in 2023 and 68 in 2024. Patients were eligible for inclusion if they had undergone a complete diagnostic workup consisting of preoperative FNA, multiparametric MRI (including DCE and DWI sequences) and subsequent surgical intervention with definitive histopathological verification. Patient age ranged from 21 to 76 years (mean 57.6 years, median 68 years). The cohort comprised 10 females (40%) and 15 males (60%). Among the 24 parotid gland lesions, 14 lesions (58.3%) were located exclusively in the superficial lobe, whereas 10 lesions (41.7%) involved both the superficial and deep lobes. No lesion was located exclusively in the deep lobe. Therefore, all parotid lesions demonstrated superficial lobe involvement. One additional lesion was located in the submandibular gland. Side distribution was right in 13 patients (52%) and left in 12 patients (48%). All patients underwent ultrasound-guided fine-needle aspiration performed by a single, experienced otolaryngology specialist to ensure procedural consistency, without Rapid On-Site Evaluation (ROSE). Conversely, studies reporting higher accuracy (82–88%) typically rely on immediate cytopathologist verification, which minimizes non-diagnostic, paucicellular yields. A 23-guage needle is typically used for the aspiration of cellular material. FNA results were interpreted according to the Milan System for Reporting Salivary Gland Cytopathology (MSRSGC) (Table 1), which standardizes salivary gland cytology reporting by dividing results into six diagnostic categories, each with an assigned risk of malignancy (ROM). In addition, DCE-MRI was performed in every case. Magnetic resonance imaging was performed using a high-field scanner with a dedicated head and neck coil. The multiparametric protocol included conventional T1- and T2-weighted sequences, followed by advanced functional imaging. For the DCE sequences, a weight-based dose of gadolinium-based contrast agent was administered. We analyzed time–intensity curves (TICs) by placing regions of interest (ROIs) within the solid, most enhancing portions of the tumors, intentionally avoiding necrotic or cystic areas. Oval ROIs were manually placed within the solid portion of each lesion, avoiding cystic, necrotic, hemorrhagic, or calcified areas, as well as visible vessels. ROI size varied depending on lesion size and morphology, with a minimum ROI area of 50 mm^2^. The slice thickness of the analyzed MRI sequences was 2 mm. The total acquisition time of the MRI protocol was 39 min and 30 s. The majority of examinations were performed on a 3.0 Tesla MRI scanner (SIGNA Pioneer, GE HealthCare; SIGNA Pioneer, GE HealthCare, Chicago, IL, USA; 23 examinations). Two examinations were performed on a 1.5 Tesla MRI scanner (MAGNETOM Sola, Siemens Healthineers MAGNETOM Sola, Siemens Healthineers, Erlangen, Germany). Thus, the MRI examinations were acquired using scanners from two different vendors. The total acquisition time of the MRI protocol was 39 min and 30 s. Three primary kinetic parameters were recorded: time to peak (TTP), washout ratio (WR), and the resulting TIC shape (Type A, B, C, or D). Additionally, DWI was employed to calculate the apparent diffusion coefficient (ADC) and the relative ADC radio (ADCr), the latter being calculated as the lesion ADC divided by the ADC of the contralateral healthy parotid gland. No imaging evidence of perineural spread was observed in any of the lesions included in this study. Therefore, perineural spread did not contribute to lesion characterization or diagnosis in our cohort. Final diagnoses were established by postoperative histopathological examination.

Statistical methods. Statistical analysis was performed to assess the association between the type of time–intensity curve (TIC) on DCE-MRI and the final histopathological diagnosis. For categorical variables, 2 × 2 contingency tables were used. Association strength was assessed by odds ratio (OR) with 95% confidence interval (95% CI). When zeros occurred in table cells, the Haldane–Anscombe correction (+0.5 to each cell was applied to allow OR estimation. Sensitivity (Se), specificity (Sp), positive predictive value (PPV), and negative predictive value (NPV) were calculated using standard diagnostic formulas. Statistical significance was assessed with Fisher’s exact test; *p* < 0.05 was considered significant.

Tumor size was measured in three anatomical axes: RL (right-left), AP (anterior–posterior), and CC (cranio-caudal). Mean lesion dimensions were 25.4 mm (range 7–110 mm) in RL, 23.1 mm (range 8–89 mm) in AP, and 27.3 mm (range 10–105 mm) in CC. Radiologically, 23 lesions were well circumscribed, while two cases demonstrated poorly defined margins. Cervical lymph node assessment showed no evidence of enlargement or pathological changes in 19 patients. Nodal evaluation was indeterminate in one case, and data were missing in five cases.

**Figure 1:**
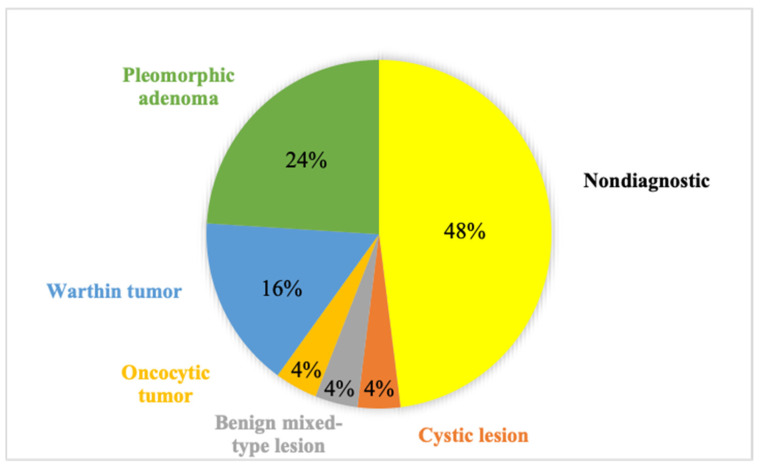
Percentage distribution of fine-needle aspiration results.

FNA was performed in all 25 patients. Non-diagnostic or indeterminate results were obtained in 12 cases (48.0%). Among 13 diagnostic FNAs, the most frequent diagnosis was pleomorphic adenoma and Warthin tumor. Single cases included cystic lesion, oncocytic tumor, and benign mixed-type lesion (Figure 1).

**Table 1:** Fine-needle aspiration results.

Milan Reporting System for Salivary Gland Cytopathology (MSRSGC)	Cytological Diagnostics	Number of Cases
I. Non-diagnostic	Non-diagnostic	12
II. Nonneoplastic	Cystic lesion	1
	Benign mixed-type lesion	1
III. Atypia of undetermined significance (AUS)		
IVa. Neoplasm: benign	Pleomorphic adenoma	6
	Warthin tumor	4
IVb. Neoplasm: salivary gland neoplasm of uncertain malignant potential (SUMP)	Oncocytic tumor	1
V. Suspicious for malignancy (SM)		
VI. Malignant		

In diffusion-weighted imaging (DWI), the apparent diffusion coefficient (ADC) and the relative apparent diffusion coefficient (ADCr, apparent diffusion coefficient ratio) were assessed. DWI-derived ADC and ADCr values showed wide intralesional variability (median ADC X × 10^−3^ mm^2^/s, IQR Y–Z). ADCr was calculated as lesion ADC divided by contralateral gland ADC. Although ADCr < 1 was observed in many lesions and may indicate relatively restricted diffusion (high cellularity), there was substantial overlap between histologic subtypes. Therefore, ADC/ADCr should be interpreted cautiously and in conjunction with other imaging and cytologic data. Further statistical analysis is warranted to define optimal thresholds.

**Figure 2:**
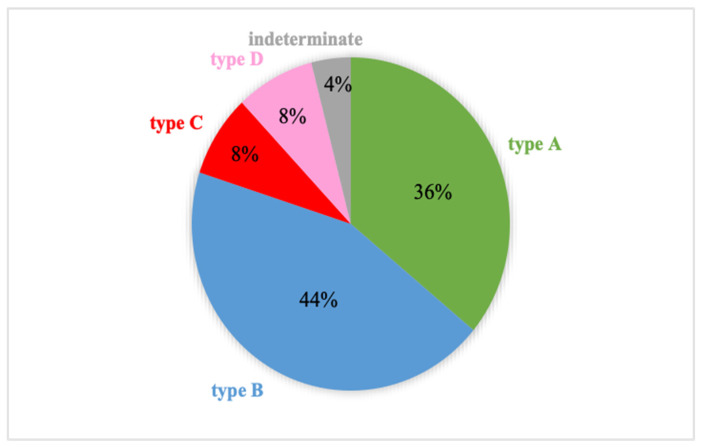
Percentage distribution of TIC types in DCE-MRI.

Dynamic contrast-enhanced MRI sequences were analyzed for time-to-peak (TTP), washout ratio (WR), and time–intensity curve (TIC) type. Mean TTP was 126.4 s (range 15.2–302 s), and mean WR was 26.8% (range 0–79.4%). These parameters reflect heterogeneous perfusion dynamics—ranging from slow, progressive signal increase in benign lesions to rapid enhancement with pronounced washout in tumors with high vascularity. Therefore, mean values for the whole cohort have limited diagnostic value. DCE parameters are most informative when analyzed according to lesion type. The most frequent TIC type was B (11 cases, 44%), followed by A (9 cases, 36%), and then C and D (2 cases each, 4% each). In one case (4%), the TIC type was indeterminate (Figure 2).

**Figure 3:**
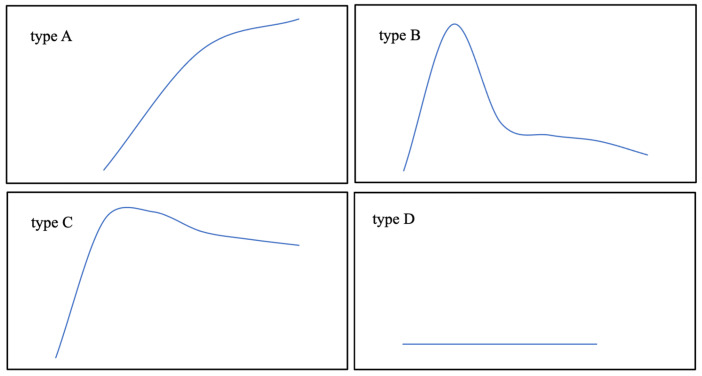
Types of time–intensity curves (TICs) according to Yabuuchi et. al. [2].

The Type A curve is characterized by a slow, progressive signal increase, likely reflecting abundant myxoid stroma and low microvessel density. In contrast, Type B features rapid enhancement followed by a washout, which serve as histological hallmarks of high vascularity and dense lymphoid stroma. Type C curves are characterized by rapid enhancement followed by washout or plateau and are most commonly associated with malignant salivary gland tumors with high vascular density. Type D curves demonstrate flat or fluctuating enhancement patterns and may occur in inflammatory, lymphoproliferative, or atypical lesions.(Table 2 and Figure 3)

**Table 2:** TIC classification in DCE-MRI.

TIC Classification	Description	Typical TTP/WR	Most Likely Diagnosis
A	slow, steady signal increase, no washout	long TTP, low WR (<30%)	most consistent with pleomorphic adenoma
B	rapid rise and rapid decline (washout)	short TTP, high WR (>60%)	most consistent with Warthin tumor
C	rapid rise followed by plateau	short TTP, moderate WR (30–60%)	often associated with malignant tumors
D	flat curve	variable TTP and WR	may indicate inflammatory changes, post-radiation changes, atypical neoplasms

Imaging reports (MRI) were available for all 25 patients. The most frequently suggested imaging diagnoses were Warthin tumor and pleomorphic adenoma. Single-case impressions (one each) included suspected malignant lesion, suspected lymphoma, suspected myoepithelial adenoma, and non-specific appearance. In one case, the MRI report did not provide a specific diagnosis (Table 3).

**Figure 4:**
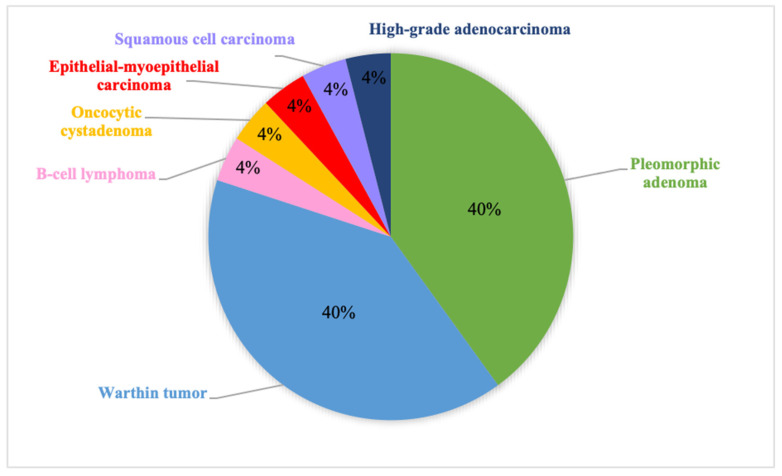
Percentage distribution of postoperative histopathologic results.

Postoperative histopathologic results were obtained for all 25 patients. The most common histologic diagnosis were Warthin tumor (10 cases, 40%) and pleomorphic adenoma (10 cases, 40%), including two pleomorphic adenomas with a predominant myoepithelial component. These two entities comprised the vast majority of histopathologic findings. Single-case diagnoses included oncocytic cystadenoma, B-cell lymphoma, epithelial–myoepithelial carcinoma, squamous cell carcinoma and a high-grade adenocarcinoma (Table 4 and Figure 4).

**Table 3:** Radiology report.

Radiology Report	Number of Cases	Percentage of Cases
Warthin tumor	10	40%
Pleomorphic adenoma	10	40%
Lymphoma	1	4%
Malignant lesion	1	4%
Myoepithelial adenoma	1	4%
Non-specific appearance	1	4%
Indeterminate	1	4%

**Table 4:** Postoperative histopathologic results.

Postoperative Histopathology	Number of Cases	Percentage of Cases
Warthin tumor	10	40%
Pleomorphic adenoma	10	40%
B-cell lymphoma	1	4%
Oncocytic cystadenoma	1	4%
Epithelial–myoepithelial carcinoma	1	4%
Squamous cell carcinoma	1	4%
High-grade adenocarcinoma	1	4%

## 3. Results

An analysis was performed comparing fine-needle aspiration results with postoperative histopathologic diagnoses. Across the whole cohort of 25 patients, FNA and final histopathology were concordant in 9 cases (36.0%) and discordant in 16 cases (64.0%). In 12 cases, the FNA results were non-diagnostic or indeterminate, preventing verification of concordance based on cytology alone. Restricting the analysis to cases with diagnostic FNA results (*n* = 13), concordance between FNA and histopathology was 9/12 (75.0%). In two cases, FNA was non-diagnostic, whereas the subsequent histopathological examination demonstrated squamous cell carcinoma in one case and B-cell lymphoma in another. In one case, FNA indicated a pleomorphic adenoma, whereas postoperative histology revealed epithelial–myoepithelial carcinoma. The highest concordance between FNA and histopathology was observed for pleomorphic adenoma.

A comparative analysis was performed between lesion characteristics on DCE-MRI and final postoperative histopathologic diagnoses in 25 patients. Radiology report was compliant with histopathology in 19 cases (76.0%), while discordance between DCE-MRI time–intensity curve (TIC) type and final histopathology was observed in 5 cases (20.0%) In one case, complete data for concordance assessment were missing. The highest agreement was seen for Warthin tumor (TIC Type B—rapid enhancement with marked washout) and pleomorphic adenoma (TIC Type A—gradual, mild signal increase). In our cohort, TIC Type C was observed in malignant lesions, including squamous cell carcinoma and epithelial–myoepithelial carcinoma, whereas TIC Type D was identified in lymphoma and atypical lesions with non-specific enhancement characteristics. Concordance was also found for cases diagnosed as lymphoma and squamous cell carcinoma. Discordance between DCE-MRI appearance and histopathology was observed for epithelial–myoepithelial carcinoma and a high-grade adenocarcinoma.

The association between TIC type on DCE-MRI and histopathologic diagnosis showed a strong statistical relationship.

The presence of a Type B curve increased the odds of a Warthin tumor 36-fold (OR = 36; 95% CI: 3.2–406.8; *p* < 0.01). Sensitivity was 90%, specificity 80%, positive predictive value (PPV) 75% and negative predictive value (NPV) 92.3%.

For pleomorphic adenomas, TIC type showed perfect concordance—100% sensitivity and 100% specificity. Ideal separation produced an infinite OR (OR → ∞). After applying the Haldance–Anscombe correction, the OR was 651 (95% CI: 11.9–35,400; *p* < 0.001) (Table 5).

These results indicate a very high diagnostic value of DCE time–intensity curve analysis for differentiating the most common benign salivary gland tumors (Appendix A).

**Table 5:** Diagnostic performance metrics of TIC types on DCE-MRI for key histopathologic diagnoses.

Diagnosis	TIC Type	OR	95% CI	Sensitivity	Specificity	PPV	NPV
Warthin tumor	Typ B	36	3.2–406.8	90%	80%	75%	92.30%
Pleomorphic adenoma	Typ A	∞ (651 *)	11.9–35,400	100%	100%	100%	100%

* OR after Haldane’a–Anscombe’a correction.

## 4. Discussion

The preoperative differentiation of salivary gland tumors remains one of the most challenging areas in head and neck pathology due to their vast morphological diversity. Our study highlights a critical clinical problem: the discrepancy between initial cytologic assessment and final histopathological outcomes. While the parotid gland is the most common site for these neoplasms, the presence of over 30 histological subtypes necessitates a diagnostic approach that is both highly sensitive and specific to guide surgical planning and preserve vital structures such as the facial nerve. Regarding the demographic distribution, our study noted a male predominance (60%), which stands in contrast to some epidemiological reports that suggest a slight female predominance in salivary gland neoplasms. This discrepancy may be attributed to the relatively small sample size of our cohort or regional variations in tumor incidence, and it underscores the importance of focusing on functional imaging biomarkers rather than purely demographic data for preoperative differentiation. Dynamic contrast-enhanced magnetic resonance imaging (DCE-MRI) is an advanced MRI technique based on analysis of contrast enhancement dynamics over time [2,15,19,20]. It permits assessment of perfusion, vascular permeability, and tissue vascularization patterns, supporting differentiation between benign and malignant lesions [6,9]. Combined with diffusion-weighted imaging (DWI), DCE-MRI provides information on cellular density and tumor microstructure, making it a valuable tool in the preoperative assessment of focal salivary gland lesions [17,18,21,22]. The integration of these functional parameters allows for a multiparametric signature that moves beyond mere anatomical description, offering insights into the physiological behavior of the tumor tissue [23,24]. By evaluating how contrast media circulates through the interstitial space, we can infer the degree of angiogenesis and capillary integrity, which are often altered in neoplastic processes. Globally and increasingly at our institution, multiparametric MRI (DCE + DWI) is actively used to resolve cases with Milan Category I (Non-diagnostic), III (Atypia of Undetermined Significance), and IVb (SUMP) cytologies. The results of our single-center analysis corroborate the growing role of MR techniques—particularly DWI and DCE-MRI—in the preoperative workup of salivary gland tumors [5,25]. In this cohort, benign lesions were predominant, with Warthin tumors and pleomorphic adenomas most frequent, consistent with the majority of published series on salivary gland neoplasms [1,26,27]. Consistent with the literature, FNA cytology demonstrates high specificity for distinguishing benign from malignant lesions but limited sensitivity, especially for indeterminate atypia (MSRSGC Category III) and lesions of uncertain malignant potential (Category IVb). In our analysis, FNA diagnostic yield was limited—concordance with postoperative histopathology was only 36%, and nearly half (48%) of FNA results were non-diagnostic or indeterminate. This high rate of inconclusive cytology often forces clinicians to perform surgeries without a clear understanding of the tumor’s nature, potentially leading to over- and under-treatment. The cytologic challenges identified in our study often arise from the presence of cystic components, extensive necrosis, or pluricellular samples, which are common in Warthin tumors and certain low-grade malignancies, leading to the non-diagnostic labels observed in 12 of our cases [11,25]. Analysis of DCE-MRI and DWI parameters showed high utility for lesion characterization with overall concordance with histopathology of 76%. Typical enhancement patterns for Warthin tumors (TIC Type B) and pleomorphic adenomas (TIC Type A) correlated with the histologic findings, supporting the potential for preliminary differentiation of common benign lesions based on perfusion and diffusion profiles [2,15,28]. Specifically, the Type A curve observed in pleomorphic adenomas—characterized by a slow, progressive increase in signal—is likely a reflection of the abundant myxoid stroma and relatively low microvessel density in these lesions. Conversely, the rapid enhancement and subsequent washout (Type B) seen in Warthin tumors are histological hallmarks of their high vascularity and dense lymphoid stroma. Our finding that a Type B curve increased the odds of a Warthin tumor 36-fold provides a powerful diagnostic indicator that can assist in distinguishing it from other lesions with 90% sensitivity and a high negative predictive value of 92.3%. These results emphasize the value of multiparametric MRI in cases where FNA is non-diagnostic or when biopsy is contraindicated due to risk of tumor seeding or facial nerve proximity. Integration of DWI and DCE-MRI data can improve surgical planning and reduce the need for additional invasive diagnostic procedures [7,18]. We observed a strong association between TIC pattern on DCE-MRI and final histopathologic diagnosis. Notably, TIC Type A exhibited perfect separation for pleomorphic adenoma in this dataset, suggesting very high specificity of this pattern in our population. However, the diagnostic complexity remains in malignant cases. For instance, the observed discordance in epithelial–myoepithelial carcinoma and high-grade adenocarcinoma in our series (where MRI was not compliant with histopathology in 20% of cases) suggests that some malignancies can mimic the perfusion profiles of benign lesions. This overlap highlights the necessity of interpreting DCE-MRI results within a comprehensive clinical context, combining them with ADC values to assess tissue cellularity. While low ADC values generally suggest malignancy due to high cellular density, the dense cellularity of Warthin tumors can also result in restricted diffusion (ADCr < 1), making the combined analysis of TIC types and ADC ratios crucial for avoiding false positives [29]. The clinical utility of our findings lies primarily in their ability to guide the tailoring of surgical interventions, particularly regarding preservation of the facial nerve and the precise adjustment of resection margins. DCE-MRI and ADC findings will not completely replace the decision to operate, as surgical resection remains the definitive treatment for persistent salivary masses to rule out malignancy and prevent local growth. Instead, these findings change practice patterns by shifting management from blind/delayed intervention to risk-stratified, proactive planning. In our practice, the management strategy is strictly differentiated based on the suspected tumor pathology. When FNA is non-diagnostic, a Type A curve (pleomorphic adenoma) alerts the surgeon to plan a formal partial parotidectomy with wide margins to avoid capsule rupture and recurrence. For pleomorphic adenomas (PAs), a watchful waiting strategy using sequential MRI is never employed. Given the well-documented risk of malignant transformation into carcinoma ex pleomorphic adenoma and the potential for continuous local growth, prompt surgical resection remains the standard of care to prevent malignant conversion. A Type B curve (Warthin tumor) in an elderly, high-risk surgical patient provides the confidence to consider active surveillance over an unnecessary, risky surgery. Conversely, a more conservative approach can be adopted for WTs due to their benign nature and negligible malignant potential. Non-operative management is increasingly considered an appropriate option, particularly for elderly asymptomatic patients or those with significant comorbidities. However, these patients are not left without surveillance; they undergo regular follow-up with clinical and ultrasound assessments every 6 to 12 months to monitor for rapid growth, secondary infection, or the development of compressive symptoms. The main limitations of this study are its retrospective, single-center design, and the relatively small sample size (*n* = 25), which severely undermines the statistical power and generalizability of the findings. Because this work was conceived as a preliminary pilot study to evaluate the feasibility of our multiparametric imaging protocol, a formal a priori power analysis was not performed. Wide coincidence intervals resulting from the small sample size limit generalizability. Consequently, the statistical power is limited. Small cohorts increase the risk of effect overestimation and favor occurrences of perfect separation that may not persist in larger series. Therefore, the exceptionally high odds ratios and diagnostic metrics reported here should be interpreted with extreme caution and treated as preliminary trends rather than definitive clinical indicators. Despite these limitations, the high ORs and diagnostic metrics observed are in line with published evidence indicating a meaningful role for DCE-MRI in salivary gland tumor differentiation. The shift toward personalized medicine in head and neck oncology requires such precise imaging biomarkers to tailor surgical interventions to the specific biological profile of the tumor. Consequently, our findings must be considered preliminary. To address these limitations, future research expanding the sample size through multi-center collaboration is required.

## 5. Conclusions

Although fine-needle aspiration is simple and widely available, its concordance with histopathology is limited, reducing its value as a standalone diagnostic tool. In our preliminary pilot cohort, dynamic contrast-enhanced MRI (DCE-MRI) combined with DWI demonstrated promising complementary value in characterizing salivary gland neoplasms, particularly when initial fine-needle aspiration results are non-diagnostic or indeterminate. Interpretation of dynamic parameters demands appropriate expertise. However, given the limited sample size of this study, these findings cannot be generalized to state the definitive superiority of imaging over cytology. Interpreting dynamic parameters requires significant expertise, and larger multi-center studies are necessary to confirm these preliminary diagnostic metrics before they can reliably guide routine clinical practice.

## Figures and Tables

**Figure A1:**
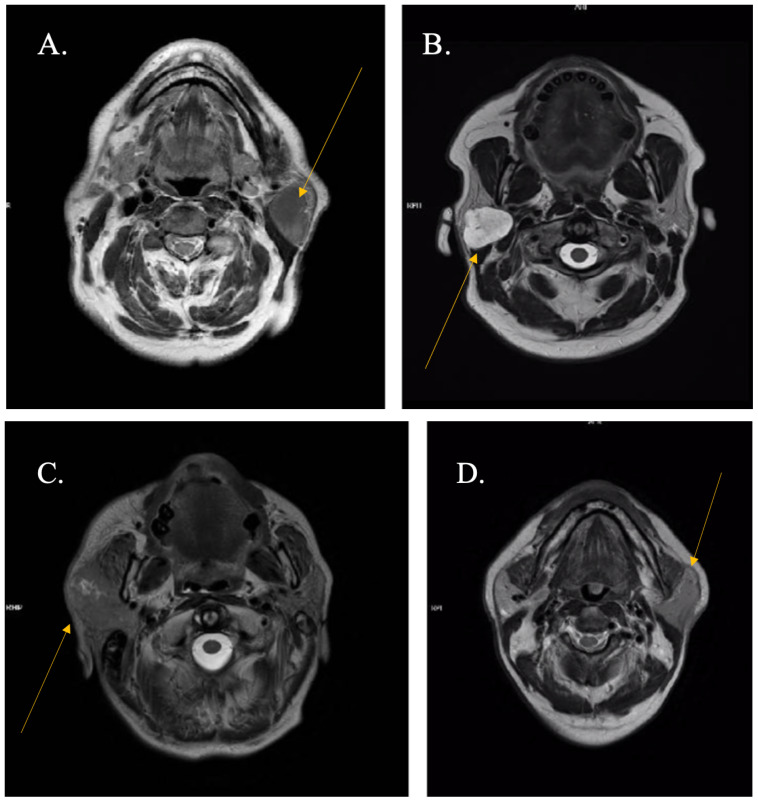
Representative MRI appearances of salivary gland tumors: (**A**) Warthin tumor. (**B**) Pleomorphic adenoma. (**C**) Squamous cell carcinoma. (**D**) Lymphoma. Yellow arrows indicate the location of the respective tumors.

## Data Availability

The data presented in this study are available on request from the corresponding author due to due to privacy and ethical restrictions.

## Abbreviations

The following abbreviations are used in this manuscript:

DCE-MRI	Dynamic contrast-enhanced magnetic resonance imaging
DWI	Diffusion-weighted imaging
FNA	Fine-needle aspiration
MSRGSC	The Milan System for Reporting Salivary Gland Cytopathology
ROM	Risk of malignancy
TIC	Time–intensity curve
TTP	Time-to-peak
WR	Washout ratio
